# Coding-Complete Genome Sequence of a *Black Queen Cell Virus* Isolate from Honey Bees (Apis mellifera) in Italy

**DOI:** 10.1128/MRA.00552-20

**Published:** 2020-07-09

**Authors:** Raied Abou Kubaa, Annalisa Giampetruzzi, Rocco Addante, Maria Saponari

**Affiliations:** a Institute for Sustainable Plant Protection, National Research Council (CNR), Bari, Italy; b Department of Soil, Plant and Food Sciences, University of Bari Aldo Moro, Bari, Italy; Queens College

## Abstract

In this study, we documented the complete coding genome sequence of a *Black queen cell virus* (BQCV) isolate from honey bees in Italy. This genome sequence illustrates a high similarity with other BQCV isolates reported worldwide and could provide insights into BQCV genome phylogeny and divergence.

## ANNOUNCEMENT

*Black queen cell virus* (BQCV) is one of the most common and widespread viral pathogens causing mortality in queen bee pupae. During a nationwide monitoring network event conducted in Italy in 2009 to 2010, 749 adult honey bee samples were analyzed by TaqMan-based real-time reverse transcription-PCR, of which 75% were infected with BQCV (1). The infected queen bee larvae turn yellow and then brown-black. The blackened areas on honeycomb cell walls that contain infected pupae give the name to this virus. BQCV was first isolated from queen prepupae and pupae found dead in their cells (2). It can be transmitted horizontally through social movements between adults within and between colonies, as well as vertically from the queen to offspring and from adults to larvae through glandular secretions, e.g., royal jelly (3). BQCV belongs to the recently established genus *Triatovirus* within the *Dicistroviridae* family and the order *Picornavirales*. The viral genome consists of a single-stranded RNA molecule approximately 8,550 nucleotides (nt) long, possessing two open reading frames (ORFs), ORF1 and ORF2, which encode polyproteins containing nonstructural and structural (capsid-forming) subunits, respectively (4). Here, we report the complete coding nucleotide sequence of an Italian BQCV isolate from infected Apis mellifera workers collected in the spring of 2018 in Puglia, South Italy.

**FIG 1:**
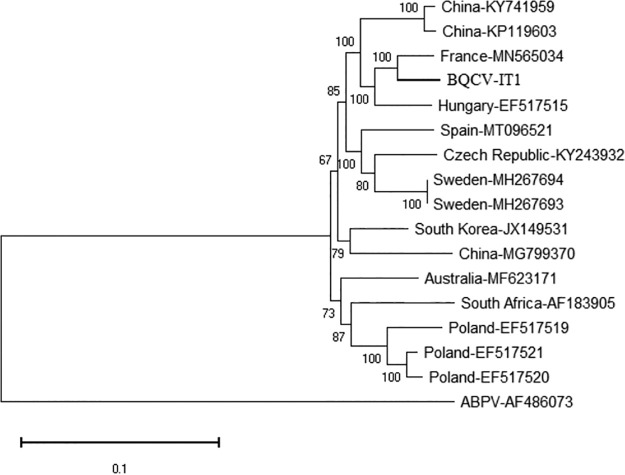
Neighbor-joining phylogenetic tree based on nucleotide sequence alignment of the whole-genome sequence of BQCV-IT1 from Italy with the currently available full-length BQCV genome sequences in GenBank. The percentage of replicate trees in which the associated taxa clustered together in the bootstrap test (1,000 replicates) is shown next to the branches. Only node values above 50% are shown. A complete genome sequence of *Acute bee paralysis virus* (ABPV; GenBank accession number AF486073) was used as an outgroup to root the tree. Evolutionary analyses were conducted in MEGA X (8).

The viral RNA was extracted from a pool of five adult honey bees collected from an apiary located in the Puglia region, all showing suspected symptoms of BQCV infection, such as dark color. The infected bees were homogenized in liquid nitrogen using a mortar and pestle, and the TRIzol (Thermo Fisher) extraction protocol and isopropanol precipitation were applied (5). The water-resuspended total RNAs were further purified using an RNeasy plant minikit (Qiagen, Valencia, CA, USA) and DNase (Promega, USA) digested following the manufacturer’s instructions. Poly(A) enrichment of the total RNAs and Illumina TruSeq RNA library construction, followed by 2 × 100-bp NovaSeq sequencing, were outsourced to Macrogen, Inc. (Seoul, South Korea). The total number of obtained reads was 36,919,326. The raw reads were quality checked using FastQC (6). Paired reads of 101 bp were assembled using metaSPAdes version 3.9.0 (7) with the “only-assembler” parameter and multiple kmers (-k, 71, 81, and 91). The coding-complete genome sequence of the BQCV isolate was assembled with an average coverage of 13.4× (3.5 reads per kilobase per million reads [RPKM]). This genome consisted of 8,458 nucleotides (nt) with a GC content of 40.30%, including 2 untranscribed regions (UTRs) at the 5′ and 3′ locations consisting of 647 nt and 154 nt, respectively. The 5′-proximal open reading frame 1 (ORF1) initiated at nucleotide position 648 and terminated at nucleotide position 5534, and the 3′-proximal ORF2 was located between nucleotide positions 5851 and 8304. The Italian BQCV isolate shared 95.93% nucleotide identity with a BQCV isolate from Vespa velutina *nigrithorax* in France (NCBI GenBank accession number MN565034) and also clustered together in the phylogenetic tree with isolates from China and Hungary (NCBI accession numbers KP119603, KY741959, and EF517515) (Fig. 1).

This is the complete coding genome sequence of BQCV-IT1, isolated from *A. mellifera* in Italy. It provides additional insights and a better understanding about BQCV genome phylogeny and divergence.

### Data availability.

The complete coding genome sequence of *Black queen cell virus* isolate BQCV-IT1 from Italy has been deposited in GenBank under the accession number MT416539. Sequencing reads are available in the SRA under BioProject accession number PRJNA637229.
